# Adaptive Induction of Growth Differentiation Factor 15 Attenuates Endothelial Cell Apoptosis in Response to High Glucose Stimulus

**DOI:** 10.1371/journal.pone.0065549

**Published:** 2013-06-14

**Authors:** Jun Li, Lijun Yang, Weijun Qin, Geng Zhang, Jianlin Yuan, Fuli Wang

**Affiliations:** 1 Department of Burns and Cutaneous Surgery, Xijing Hospital, Fourth Military Medical University, Xi’an, China; 2 Department of Urology, Xijing Hospital, Fourth Military Medical University, Xi’an, China; National Center for Scientific Research Demokritos, Greece

## Abstract

Growth differentiation factor 15 (GDF15), a direct target gene of p53, is a multifunctional member of the TGF-β/BMP superfamily. GDF15 can be induced and is implicated as a key secretory cytokine in response to multiple cellular stimuli. Accumulating evidence indicates that GDF15 is associated with the development and prognosis of diabetes mellitus, while whether GDF15 can be induced by high glucose is unknown. In the present study, we revealed that high glucose could induce GDF15 expression and secretion in cultured human umbilical vein endothelial cells in a ROS- and p53-dependent manner. Inhibition of high glucose-induced GDF15 expression by siRNA demonstrated that adaptively induced GDF15 played a protective role against high glucose-induced human umbilical vein endothelial cell apoptosis via maintaining the active state of PI3K/Akt/eNOS pathway and attenuating NF-κB/JNK pathway activation. The protective effects of GDF15 were probably achieved by inhibiting ROS overproduction in high glucose-treated human umbilical vein endothelial cells in a negative feedback manner. Our results suggest that high glucose can promote GDF15 expression and secretion in human umbilical vein endothelial cells, which in turn attenuates high glucose-induced endothelial cell apoptosis.

## Introduction

Growth differentiation factor 15 (GDF15), also known as macrophage inhibiting cytokine 1 (MIC-1) [1] [2], placental transformation growth factor (PTGF-β) [3] [4] [5], prostate derived factor (PDF) [6], placental bone morphogenetic protein (PLAB) [7] [8], NSAID activated gene-1 (NAG-1) [9] [10], PL74 [11], is a multifunctional member of the transforming growth factor beta (TGF-β)/bone morphogenetic protein (BMP) superfamily, which is involved in the regulation of cell proliferation, differentiation, apoptosis, inflammation, and tumorigenesis. Under physiological conditions, GDF15 is highly expressed in prostate and placenta, while it is weakly expressed in most other tissues [12]. GDF15 expression can be dramatically induced and is implicated as a key secretory cytokine in response to multiple cellular stressors, such as acute injury, inflammation and cancer [13]. In cardiovascular disease, GDF15 has great potential as a biomarker for prognosis and as a protective cytokine against different stimuli via specific signaling pathways, such as PI3K/Akt, ERK1/2, and SMAD2/3 [14] [15] [16] [17] [18] [19].

Endothelial cell dysfunction plays a critical role in endothelial cell activation and atherosclerotic plaque formation. Diabetes mellitus is a major risk factor for cardiovascular disease and is the leading cause of vascular complications such as atherosclerosis [20]. Accumulating evidence implicates that hyperglycemia plays a central role in the pathogenesis of microvascular complications [21]. The etiology of diabetic atherosclerosis includes the increase of reactive oxygen species (ROS) and the decrease of nitric oxide (NO) bioavailability in endothelial cells as a result of high glucose level [22]. ROS, such as superoxide anion (O_2_^−^), hydrogen peroxide (H_2_O_2_), and hydroxyl radical (HO•), consist of radical and non-radical oxygen species formed by the partial reduction of oxygen. ROS can function as signaling molecules and participate in the regulation of cell activities at lower concentrations, but at higher concentrations ROS can induce oxidative stress that causes cellular injury and death [23] [24]. Much evidence implicates that high glucose-induced apoptosis in human endothelial cells is associated with increased ROS concentrations and subsequent triggered multiple signaling pathways [25] [26] [27] [28] [29] [30] [31].

The tumor suppressor protein p53 is a redox active transcription factor that can interact with ROS indirectly through signaling networks or directly through redox-sensitive thiol groups (–SH) of cysteines (Cys) located in the DNA-binding domain of p53. ROS play a central role in redox signaling and can act as both an upstream signal triggering p53 activation and a downstream factor leading to cell apoptosis [32]. Researches concerning the role of p53 in endothelial cell dysfunction are less. Some reports have focused on p53 activation in human endothelial cells under high glucose culture condition, indicating that p53 is involved in high glucose-induced cellular senescence and contributes to diabetic atherosclerosis [33] [34] [35].

In view of increased GDF15 expression in response to multiple stimuli, we raised the hypothesis that GDF15 expression might be induced in high glucose-treated endothelial cells. The present study is to test this hypothesis and clarify its underlying mechanisms.

## Materials and Methods

### Cell Culture

Human umbilical venous endothelial cells (HUVECs) were purchased from American Type Culture Collection (ATCC, Rockville, MD, USA). The cells were cultured in ATCC-formulated of F-12K medium, supplemented with 0.1 mg/ml heparin, 0.05 mg/ml endothelial cell growth supplement (Sigma-Aldrich Co., St Louis, MO, USA), 10% fetal bovine serum (Gibco, Grand Island, NY, USA), 1% penicillin-streptomycin (Gibco).

### Western Blotting

The cells were washed twice with precooled PBS and then lysed in RIPA buffer. After centrifugation at 4°C, the supernatant was collected and the protein concentration was measured using the BCA protein assay kit (Pierce, Rockford, IL, USA). 50–80 µg of total protein extract was resolved by SDS-PAGE and transferred to nitrocellulose membranes. The membranes were blocked in 5% milk and probed with primary antibodies overnight at 4°C. Anti-GDF15 rabbit monoclonal antibody (mAb), anti-β-actin rabbit polyclonal antibody (pAb), anti-phospho-PI3K p85 (Tyr458)/p55 (Tyr199) rabbit pAb, anti-PI3K p85 rabbit pAb, anti-phospho-Akt (Ser473) rabbit mAb, anti-Akt rabbit mAb, anti-phospho-eNOS (Ser1177) rabbit pAb, anti-eNOS rabbit pAb, anti-phospho-ERK1/2 (Thr202/Tyr204) rabbit mAb, anti-ERK1/2 rabbit pAb, anti-phospho-Smad2 (Ser465/467)/Smad3 (Ser423/425) rabbit mAb, anti-smad2/3 rabbit pAb, and anti-cleaved caspase-3 rabbit mAb were purchased from Cell Signaling Technologies (Beverly, MA, USA). Anti-p53 rabbit pAb and anti-p21 rabbit pAb were were purchased from Santa Cruz Biotechnology (Santa Cruz, CA, USA). The next day, membranes were washed with 1xTBST, and incubated with horseradish peroxidase (HRP)-conjugated goat anti-rabbit IgG (Santa Cruz, CA, USA) for 1 h at room tempreture. Immunoreactive proteins were detected with an enhanced chemiluminescence detection system (Amersham Life Science, Arlington Heights, IL, USA). Bands were quantitated by ImageJ (National Institute of Health, USA) and the fold expression was indicated as the relative protein level.

### Real-time PCR (RT-PCR)

RT-PCR technique was used to analyze mRNA expression of GDF15 in HUVEC cells. Briefly, total RNA was extracted using the TRIZOL reagent according to the manufacturer’s instructions (Invitrogen, Carlsbad, CA). RT-PCR for GDF15 was performed using One Step SYBR® PrimeScript® RT-PCR Kit II (TaKaRa Biotechnology, Dalian, China) with forward primer 5′-GACCCTCAGAGTTGCACTCC-3′ and reverse primer 5′-GCCTGGTTAGCAGGTCCTC-3′. GAPDH was used as an internal control. Primers of GAPDH was forward 5′-TGTGGGCATCAATGGATTTGG-3′ and reverse 5′-ACACCATGTATTCCGGGTCAAT-3′. Amplification and detection were performed with ABI Prism 7500 RT-PCR System (Applied Biosystems). Amplicons were analyzed using the ΔΔCt method.

### ELISA Assay

The culture supernatant was measured by ELISA using RayBio® human GDF15 ELISA kit (RayBiotech Inc., Norcross, GA, USA) according to the manufacturer’s protocol. In brief, 100 µl of each standard and sample were added into appropriate wells. After incubating for 2.5 hours, the solution was discarded and washed 4 times with wash solution. Then 100 µl of biotinylated antibody was added to each well and incubated for 1 hour at room temperature. Afer 4 times wash, 100 µl of streptavidin solution was added to each well and incubated for 45 minutes. Afer 4 times wash, 100 µl of TMB one-step substrate reagent was added to each well and incubated for 30 minutes at room temperature in the dark. Finally, 50 µl of stop solution was to each well. Absorbance at 450 nm was read immediately using an microplate reader.

### Intracellular ROS Detection

For experiments including ROS inhibitors, the cells were incubated with 10 µmol/l NAD(P)H oxidase (NOX) and NOS inhibitor diphenyleneiodonium (DPI) (Sigma-Aldrich, St Louis, MO, USA), or 1 mmol/l superoxide dismutase mimetic TEMPOL (Sigma-Aldrich). Measurements of intracellular ROS levels were performed using 2′,7′-dichlorofluorescein diacetate (DCFH_2_-DA, Molecular Probes, Eugene, OR, USA). Cell samples were incubated in the presence of 50 µM DCFH_2_-DA in phosphate buffered saline (PBS) at 37°C for 30 min, then washed with PBS and centrifuged at 1200 rpm to remove the extracellular DCFH_2_-DA. After incubation, cells were resuspended in ice-cold PBS for flow cytometry analysis.

### RNA Interference

p53 siRNA, GDF15 siRNA, and control siRNA products were purchased from Santa Cruz Biotechnology. Transfection of siRNA was performed using X-treme GENE siRNA transfection reagent (Roche Applied Science, Prague, Czech Republic) according to the manufacturer’s instructions. 12 h after transfection, HUVEC cells were used for experiments.

### Apoptosis Assay by Flowcytometry (FCM)

After GDF15 siRNA transfection, HUVEC cells were treated with high glucose for different time period. Then HUVEC cells were harvested and incubated with 0.2 µg/ml Annexin V-FITC (Pharmingen, San Diego, California, USA) and 50 µg/ml propidium iodide (PI) (Pharmingen) for 15 min at room temperature. After that, HUVEC cell apoptosis was analysed by flowcytometry.

### Nitric Oxide (NO) Colorimetric Assay

NO is rapidly oxidized to nitrite and nitrate which are used to quantitate NO production. The concentrations of NO in the culture medium were measured using the nitric oxide colorimetric assay kit (BioVision, Mountain View, CA) according to the manufacturer’s protocol. Briefly, the nitrate was converted to nitrite utilizing nitrate reductase, then the nitrite was converted to a deep purple azo compound using Griess reagent. The amount of the azochromophore was detected by the colorimetric determination at 540 nm using a microplate reader.

### Determination of NF-κB Activity

Transactivation activity of NF-κB in HUVEC cells was monitored using a Cignal NF-κB reporter (luc) kit (Qiagen, Valencia, CA, USA). The reporter contains a mixture of inducible NF-κB-responsive firefly luciferase construct and constitutively expressing Renilla luciferase construct (40∶1). The negative control reporter contains a mixture of non-inducible firefly luciferase construct and constitutively expressing Renilla luciferase construct (40∶1). NF-κB-responsive luciferase activity was measured by a a dual luciferase reporter assay system (Promega Corp., Madison, WI).

### JNK Activity Assay

c-Jun N-terminal kinase (JNK) activity was detected using a JNK activity assay kit (Abnova, Walnut, CA, USA) according to the manufacturer’s instructions. The kit utilizes a JNK-specific antibody and the protein A-Sepharose to immunoprecipitate JNK from cell lysate. Activity of the JNK is then determined in a kinase reaction using recombinant c-Jun as substrate. Phosphorylation of the c-Jun can be analyzed by western blot analysis using the rabbit anti-phospho-c-Jun (Ser73) specific antibody at 1∶1000 dilutions.

### Statistical Analysis

All experiments were performed at least three times. Results were presented as mean ± standard deviation (SD). Statistical analysis of the data was performed by one-way analysis of variance (ANOVA). Values of *P*<0.05 were considered to be statistically significant.

## Results

### GDF15 is Induced in HUVEC Cells in Response to High Glucose

**Figure 1:**
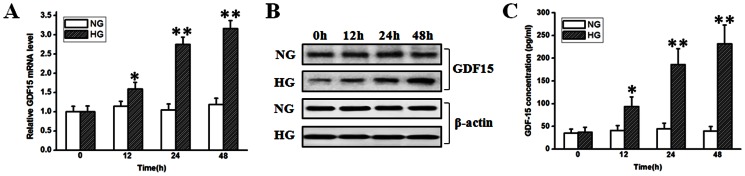
Adaptive induction of GDF15 in HUVEC cells in response to high glucose stimulus. HUVEC cells were treated with normal (5.5 mmol/l) or high (33.3 mmol/l) glucose. At indicated time points, GDF15 expression and secretion were detected by RT-PCR, western blot, and ELISA. A, analysis of GDF15 mRNA expression with RT-PCR. B, western blots of GDF15 and β-actin. C, ELISA assay of secreted GDF15. NG, normal glucose. HG, high glucose. *, *P*<0.05, **, *P*<0.01 versus NG.

Under physiological conditions, GDF15 is weakly expressed in most tissues including the heart. In response to pathological or environmental stress, however, GDF15 expression may sharply increase [36]. To test whether GDF15 can be induced in response to high glucose, we treated HUVEC cells with normal (5.5 mmol/l) or high (33.3 mmol/l) glucose and detected the changes of GDF15 expression. At indicated time point, GDF15 expression in HUVEC cells was monitored by RT-PCR and western blot. Secreted GDF15 in the culture medium was detected using ELISA assay. As shown in Fig. 1 A and B, GDF15 mRNA and protein were induced by high glucose, displaying significant upregulation at 24 h compared with the control, and the tendency was maintained at 48 h. The ELISA assay showed that secreted GDF15 in the culture medium also increased at 24 and 48 h (Fig. 1 C). These results demonstrate that high glucose can induce GDF15 expression and secretion in HUVEC cells in a time-dependent manner.

### Adaptive Induction of GDF15 Expression is ROS-dependent

**Figure 2:**
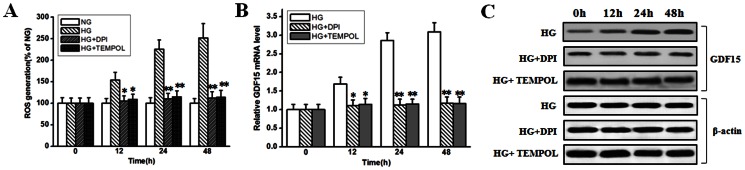
ROS inhibitors abolished high glucose-induced GDF15 expression. HUVEC cells were treated with normal (5.5 mmol/l) or high (33.3 mmol/l) glucose in the absence or presence of 10 µmol/l DPI or 1 mmol/l TEMPOL. A, ROS production were assayed as described in the materials and methods. B, C, at indicated time points, GDF15 mRNA and protein expression were detected by RT-PCR and western blot. NG, normal glucose. HG, high glucose. *, *P*<0.05, **, *P*<0.01 versus HG.

The vascular complications in diabetes are causally associated with hyperglycemia-induced ROS overproduction and a large body of evidence has suggested that endothelial dysfunction is caused by ROS [22]. Thus, adaptive induction of GDF15 expression in response to high glucose might be due to ROS overproduction. For this purpose, we treated HUVEC cells with 10 µmol/l NOX and NOS inhibitor DPI, which is chiefly responsible for ROS production under high-glucose conditions [37], or 1 mmol/l superoxide dismutase mimetic TEMPOL, then high glucose-induced ROS overproduction and GDF15 expression were assayed. As shown in Fig. 2 A, in the presence of DPI or TEMPOL, high glucose-induced ROS overproduction was restored to a level approaching that observed in control cells. As shown in Fig. 2 B and C, when high glucose-induced ROS overproduction was inhibited by DPI, adaptive induction of GDF15 expression was abolished. The same results were obtained when using TEMPOL. Taken together, these results suggest a key role for ROS in mediating high glucose-induced increase of GDF15 expression in HUVEC cells.

### Adaptive Induction of GDF15 Expression is p53-dependent

**Figure 3:**
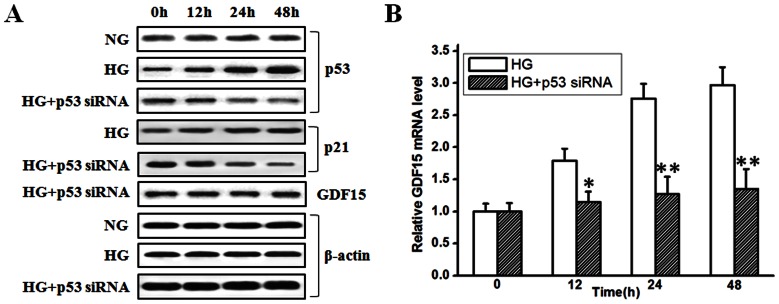
Knockdown of p53 expression inhibited high glucose-induced GDF15 expression. p53 siRNA was transfected into HUVEC cells. 12 h after transfection, HUVEC cells were treated with high glucose (33.3 mmol/l). A, at indicated time points, p53, p21, GDF15, and β-actin proteins were detected by western blot. B, GDF15 mRNA was detected by RT-PCR. NG, normal glucose. HG, high glucose. *, *P*<0.05, **, *P*<0.01 versus HG.

GDF15, containing two p53 binding sites in its promotor region, is a direct target gene of p53. DNA damage and hypoxia can induce GDF15 expression in a p53-dependent manner [38]. p53 accumulation and activation have been implicated in high glucose-induced HUVEC cell dysfunction [33] [34] [35]. Therefore, high glucose-induced GDF15 expression in HUVEC cells might be also mediated in a p53-dependent pathway. To this end, siRNA to p53 was used to knockdown p53 expression in HUVEC cells. p53 and p21 expression were detected to test the efficiency of siRNA to p53. As shown in Fig. 3 A, high glucose could induce p53 accumulation in HUVEC cells in a time-dependent manner. Transfection of p53 siRNA into HUVEC cells significantly restored high glucose-induced p53 accumulation to a level even lower than that observed in control cells. p21 expression was monitored as an indicator of functional p53. High glucose-induced p53 accumulation was associated with increased p21 expression, and knockdown of p53 by siRNA was associated with decreased p21 expression. When high glucose-induced p53 accumulation was inhibited by siRNA to p53, high glucose-induced GDF15 expression was also inhibited (Fig. 3 A and B). Transfection of negative control siRNA into HUVEC cells had no effects on p53, p21, and GDF15 expression (data not shown). Taken together, these results suggest that adaptive induction of GDF15 expression is dependent on p53 activation in high glucose-treated HUVEC cells.

### High Glucose Induced-HUVEC Cell Apoptosis is Attenuated by Adaptively Induced GDF15 Expression

**Figure 4:**
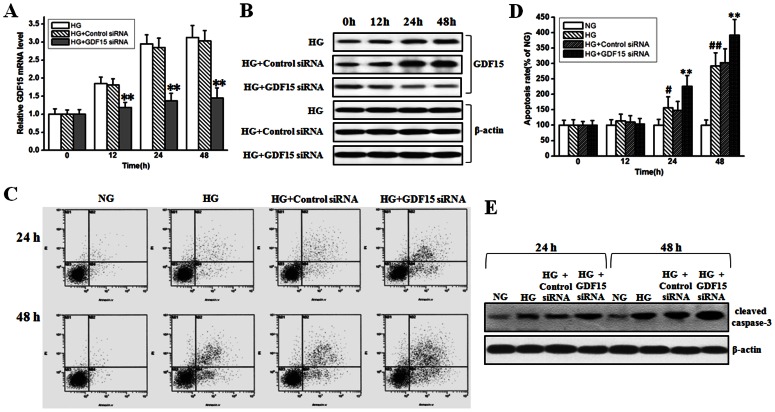
Inhibition of adaptively induced GDF15 expression enhanced high glucose-induced HUVEC cell apoptosis. GDF15 siRNA or negative control siRNA were transfected into HUVEC cells. 12 h after transfection, HUVEC cells were treated with normal (5.5 mmol/l) or high (33.3 mmol/l) glucose. A, B, mRNA and protein levels of GDF15 was detected to test the efficiency of GDF15 siRNA. C, D, FCM results and statistical analysis of high glucose-induced HUVEC cell apoptosis with or without GDF15 siRNA transfection. E, high glucose-induced caspase-3 cleavage with or without GDF15 siRNA transfection. NG, normal glucose. HG, high glucose. *, *P*<0.05, **, *P*<0.01 versus HG. ^#^, *P*<0.05, ^##^, *P*<0.01 versus NG.

Diabetes mellitus causes multiple cardiovascular complications. High glucose can induce ROS generation and apoptosis in endothelial cells [25] [26] [27] [29]. GDF15 is seen as a protective cytokine against multiple stimuli in cardiovascular disease [14] [15] [16] [17] [18] [19]. To test whether increased GDF15 secretion can play a protective role against high glucose-induced HUVEC cell apoptosis, GDF15 siRNA was transfected into HUVEC cells, then HUVEC cell apoptosis was measured by FCM, cleaved caspase-3 was also detected by western blot. As shown in Fig. 4 A and B, GDF15 siRNA effectively inhibited high glucose-induced GDF15 expression in mRNA and protein levels at 24 and 48 h. High glucose could cause HUVEC cell apoptosis at 24 and 48 h, and interestingly, knockdown of high glucose-induced GDF15 expression further enhanced cell apoptosis rate (Fig. 4 C and D). Cleaved caspase-3 increased after high glucose treatment, and GDF15 siRNA further enhanced caspase-3 cleavage (Fig. 4 E). Transfection of negative control siRNA into HUVEC cells had no effects on GDF15 expression, cell apoptosis, and caspase-3 cleavage (Fig. 4). These results demonstrate that high glucose-induced GDF15 expression plays a protective role against HUVEC cell apoptosis.

### Adaptive Induction of GDF15 Maintains the Active State of PI3K/Akt/eNOS Pathway

**Figure 5:**
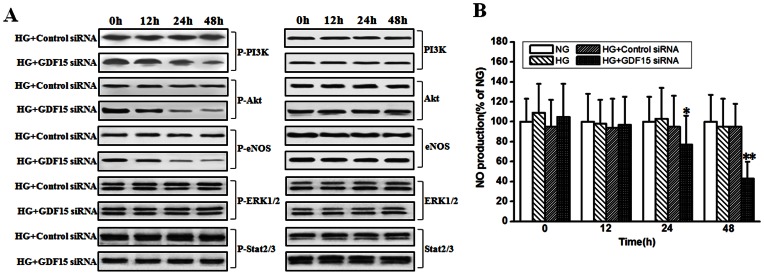
Inhibition of adaptively induced GDF15 expression attenuated PI3K/Akt/eNOS pathway. GDF15 siRNA or negative control siRNA were transfected into HUVEC cells. 12 h after transfection, HUVEC cells were treated with high (33.3 mmol/l) glucose. A, at indicated time points, phosphorylation and total amount of PI3K, Akt, eNOS, ERK1/2, and Stat2/3 were detected by western blot. B, The concentrations of NO in the culture medium were measured using the nitric oxide colorimetric assay kit as described in the materials and methods. NG, normal glucose. HG, high glucose. *, *P*<0.05, **, *P*<0.01 versus HG.

The PI3K/Akt, ERK1/2, and SMAD2/3 signaling pathways are involved in the cardioprotective effects of GDF15 [36]. Much evidence shows that the PI3K/Akt/eNOS signaling pathway plays a protective role against ROS-induced endothelial cell injury. Attenuation of PI3K/Akt/eNOS signaling pathway is implicated in high glucose-induced HUVEC cell apoptosis [25]. We speculated that adaptive induction of GDF15 could protect HUVEC cell against high glucose-induced apoptosis by activating PI3K/Akt/eNOS pathway. As shown in Fig. 5 A, when adaptive induction of GDF15 was inhibited by GDF15 siRNA, PI3K, Akt, and eNOS phosphorylation were attenuated more rapidly in HUVEC cells, compared with negative control siRNA. Next, we investigated the effects of GDF15 siRNA on NO production. As shown in Fig. 5 B, NO production was significantly lower in GDF15 siRNA-transfected HUVEC cells than in negative control siRNA-transfected cells. ERK1/2 and SMAD2/3 phosphorylation were not affected by GDF15 siRNA in HUVEC cells. These results suggest that high glucose-induced GDF15 expression in HUVEC cells can activate PI3K/Akt/eNOS pathway, which in turn plays a protective role against HUVEC cell apoptosis.

### Adaptive Induction of GDF15 Attenuates NF-κB/JNK Pathway Activation

**Figure 6:**
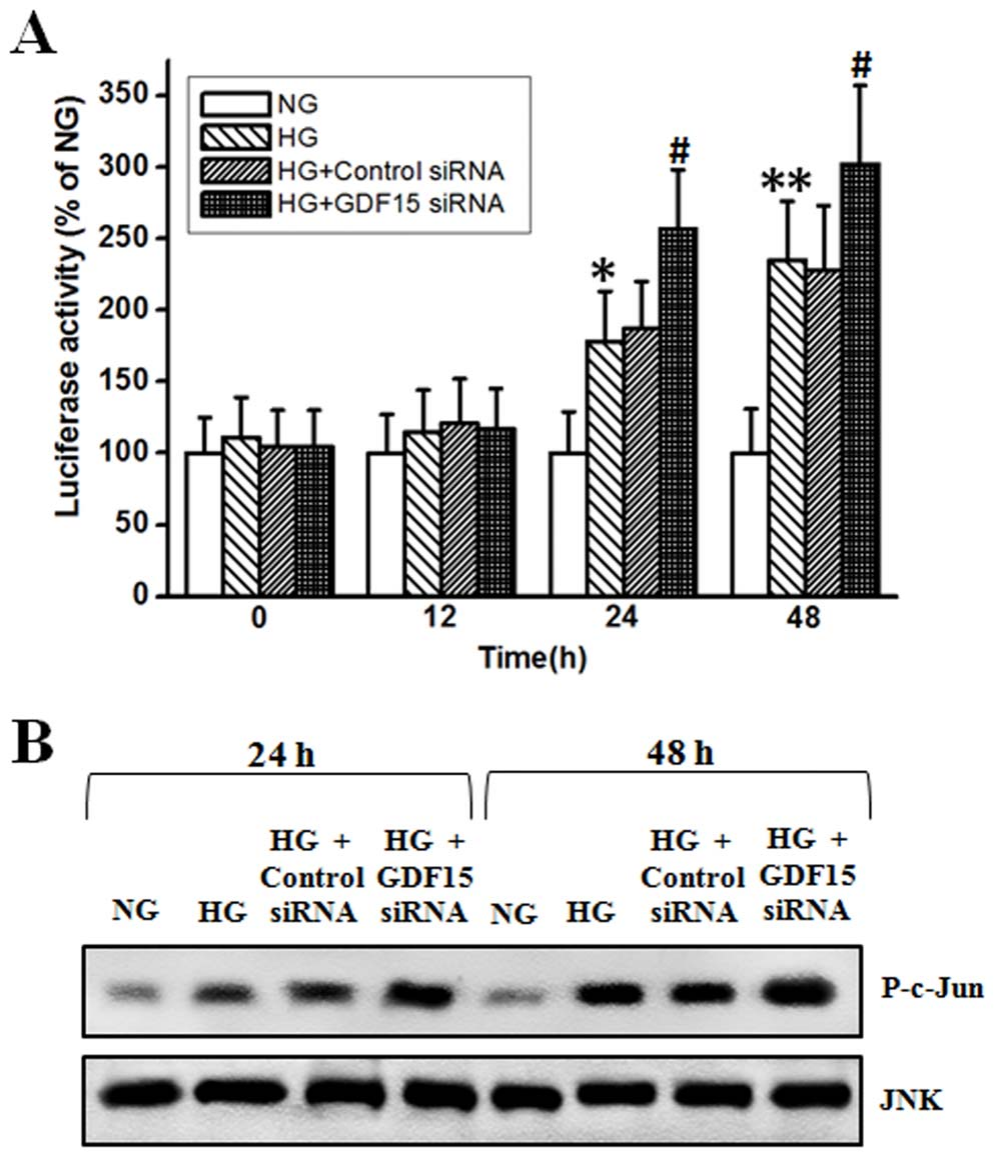
Inhibition of adaptively induced GDF15 expression promoted NF-κB/JNK activation. GDF15 siRNA or negative control siRNA were transfected into HUVEC cells. 12 h after transfection, HUVEC cells were treated with normal (5.5 mmol/l) or high (33.3 mmol/l) glucose. A, at indicated time points, NF-κB luciferase activity was detected using a Cignal NF-κB reporter (luc) kit as described in the materials and methods. B, JNK activity was detected by a JNK activity assay kit using recombinant c-Jun as substrate as described in the materials and methods. NG, normal glucose. HG, high glucose. *, *P*<0.05, **, *P*<0.01 versus NG. ^#^, *P*<0.05 versus HG.

Activation of NF-κB can prevent apoptosis in many cells types by down-regulation of JNK signaling. However, accumulating evidence implicates that ROS-mediated activation of NF-κB/JNK pathway plays an important role in high glucose-induced HUVEC cell apoptosis [25] [26] [27] [39]. To test whether high glucose-mediated activation of NF-κB/JNK pathway can be affected by adaptively induced GDF15, GDF15 siRNA was transfected into HUVEC cells, then the activity of NF-κB and JNK were assayed. As shown in Fig. 6 A and B, NF-κB luciferase activity and JNK activity were significantly higher in high glucose-treated HUVEC cells, compared with normal glucose-treated HUVEC cells. When GDF15 siRNA was transfected into HUVEC cells, NF-κB luciferase activity and JNK activity were further enhanced, compared with negative control siRNA. These results demonstrate that high glucose-induced GDF15 expression can attenuate NF-κB/JNK activation in HUVEC cells.

### Adaptively Induced GDF15 Negatively Regulates ROS Generation

**Figure 7:**
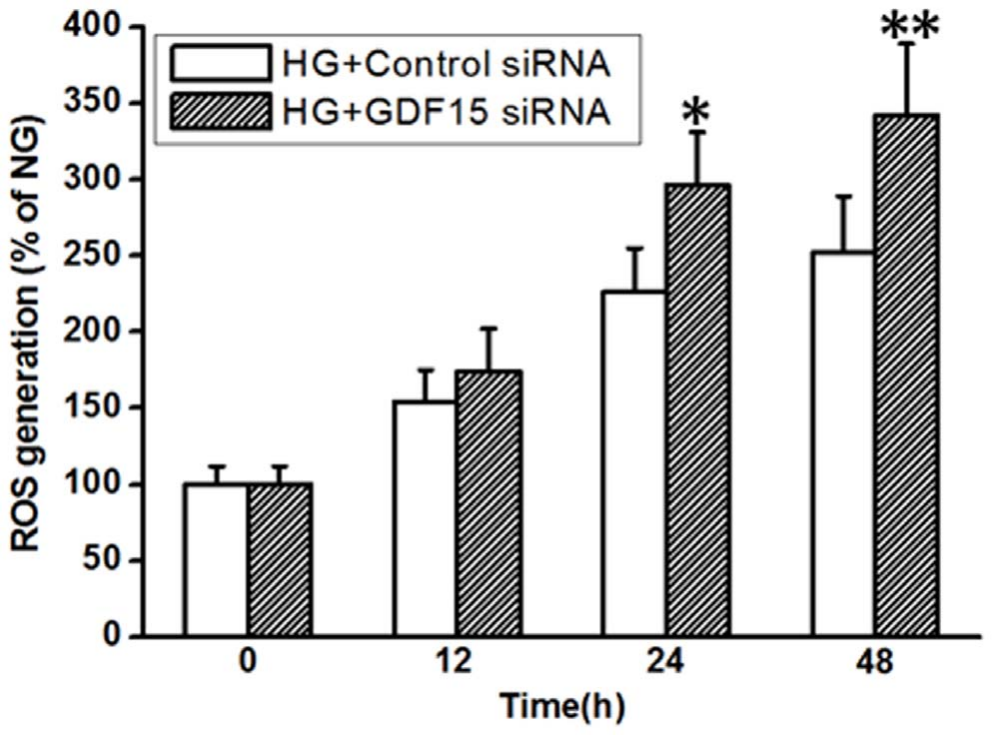
Inhibition of adaptively induced GDF15 expression increased ROS production. GDF15 siRNA or negative control siRNA were transfected into HUVEC cells. 12 h after transfection, HUVEC cells were treated with high (33.3 mmol/l) glucose. At indicated time points, ROS production were assayed as described in the materials and methods. HG, high glucose. *, *P*<0.05, **, *P*<0.01 versus Control siRNA.

Overproduction of ROS in mitochondria and subsequent activation of many signaling pathways play a fundamental role in hyperglycemia-induced vascular complications [22]. Increased ROS generation can cause HUVEC cell apoptosis by inhibiting of PI3K/Akt/eNOS/NO pathway and activation of NF-κB/JNK/caspase-3 pathway [25]. RNA interference of GDF15 expression further enhanced high glucose-induced HUVEC cell apoptosis (Fig. 4 C and D), therefore, we speculated that adaptive induction of GDF15 might down-regulate ROS production in a negative feedback manner. As shown in Fig. 7, under high glucose culture condition, when GDF15 siRNA was transfected into HUVEC cells, ROS production significantly increased, compared with negative control siRNA. The results suggest that adaptively induced GDF15 expression mediates a negative feedback regulation of ROS generation in high glucose-treated HUVEC cells.

## Discussion

In the present study, we discovered that high glucose could induce GDF15 expression and secretion in HUVEC cells in a ROS- and p53-dependent manner. Adaptively induced GDF15 protected against high glucose-induced HUVEC cell apoptosis via maintaining the active state of PI3K/Akt/eNOS pathway and attenuating NF-κB/JNK pathway activation, which might be achieved by down-regulation of ROS generation in high glucose-treated HUVEC cells. To the best of our knowledge, this is the first study to indicate that GDF15 can be induced by high glucose, which in turn protects endothelial cells against high glucose-induced apoptosis.

GDF15 is a multifunctional member of the TGF-β/BMP superfamily. In addition to prostate and placenta tissues, GDF15 is weakly expressed in most tissues under physiological conditions, while GDF15 expression can be induced in disease states [12] [13]. For example, GDF15 is induced and seen as a biomarker in patients with heart disease [36]. Recently, evidence indicates that circulating GDF15 concentration is closely associated with the development and prognosis of diabetes mellitus, and that overexpression of GDF15 can improve glucose tolerance [40] [41]. Hyperglycemia is one of the classic symptoms of diabetes mellitus and hyperglycemia-induced endothelial cell apoptosis plays an important role in vascular complications such as diabetic atherosclerosis [20] [21]. GDF15 expression is increased in the lung of patients with pulmonary arterial hypertension and predominantly locates in pulmonary microvascular endothelial cells [19], while whether GDF15 can be induced in endothelial cells under hyperglycemic conditions is unknown. In the present study, using an *in vitro* model, we discovered that high glucose could induce GDF15 expression and secretion in HUVEC cells in a time-dependent manner (Fig. 1). In patients with obesity, expression of GDF15 can be induced by very low calorie diet (VLCD) [40], which seems counter to the findings of the present study. Anti-inflammatory drugs such as the nonsteroidal anti-inflammatory drugs (NSAID) can induce GDF15 expression [13], and VLDC can significantly reduce low-grade inflammation of type 2 diabetes mellitus patients [42]. Thus, it is possible that VLCD-induced GDF15 expression might be mediated by reducing inflammation.

Endothelial cells are unable to regulate passive inflow of glucose in a hyperglycaemic environment. Excessive glucose metabolism ultimately promotes the production of ROS, which are able to decline the endothelial antioxidant systems, directly damage multiple biomolecules, increase lipid peroxidation and develop the insulin resistance in diabetes [23] [24]. Thus, ROS plays a central role in hyperglycemia-induced endothelial cell injury. N-(4-hydroxyphenyl)-retinamide (4HPR) treatment of A2780 ovarian cancer cells can induce PLAB/GDF15 upregulation via a ROS-dependent mechanism [8]. Here, we showed that high glucose-induced GDF15 expression in HUVEC cells was also ROS-dependent (Fig. 2). GDF15 is a direct target gene of p53, while induction of GDF15 expression includes both p53-dependent and p53-independent mechanisms [5] [38]. In glioblastoma cell lines, hypoxia-induced GDF15 up-regulation is mediated through a p53 and hypoxia inducible factor 1 (HIF-1) independent pathway [43]. In the present study, inhibition of high glucose-induced p53 accumulation by p53 siRNA abolished GDF15 induction, indicating that adaptively induced GDF15 expression by high glucose in HUVEC cells was p53-dependent (Fig. 3).

There are many contradictory results with regard to the role of GDF15 in cancer cells [2]. The majority of these studies have suggested that GDF15 is a proapoptotic molecule promoting tumor cell apoptosis [44] [45] [46] [47], while other studies suggest that GDF15 may facilitate tumor progression [48] [49] [50] [51]. In cardiovascular disease, much evidence demonstrates that GDF15 is a protective cytokine against multiple stimuli via many signaling pathways including PI3K/Akt, ERK1/2, and SMAD2/3 [36]. Here, we reported that adaptive induction of GDF15 played a protective role against high glucose-induced HUVEC cell apoptosis via PI3K/Akt/eNOS pathway, but not ERK1/2 and SMAD2/3 pathways (Fig. 4 and 5). ROS-mediated activation of NF-κB/JNK pathway mediates high glucose-induced HUVEC cell apoptosis [25] [26] [27] [39]. It is reported that GDF15 can inhibit differentiation of RAW264.7 macrophages into osteoclasts by suppressing NF-κB activity via delaying IκB degradation [52]. We found that adaptively induced GDF15 by high glucose attenuated NF-κB/JNK activation (Fig. 6). ROS plays a critical in hyperglycemia-induced vascular complications [22], and it is reported that GDF15 can protect cultured cerebellar granule neurons (CGN) against low potassium-induced cell apoptosis via attenuating ROS formation [53], thus we speculated that the protective role of adaptively induced GDF15 expression might be associated with ROS formation in high glucose-treated HUVEC cells. When high glucose-induced GDF15 expression was inhibited by siRNA, ROS production significantly increased in HUVEC cells (Fig. 7).

In summary, our current study suggests that high glucose can promote GDF15 expression and secretion in HUVEC cells, which can attenuate high glucose-induced cell apoptosis in a negative feedback manner. These results need to be further elucidated using diabetic animal models.
